# Effect of Birch Sap as Solvent and Source of Bioactive Compounds in Casein and Gelatine Films

**DOI:** 10.3390/membranes13090786

**Published:** 2023-09-11

**Authors:** María Carpintero, Ismael Marcet, María Zornoza, Manuel Rendueles, Mario Díaz

**Affiliations:** Department of Chemical and Environmental Engineering, University of Oviedo, C/Julian Clavería 8, 33006 Oviedo, Spain; carpinteromaria@uniovi.es (M.C.); marcetismael@uniovi.es (I.M.); mariodiaz@uniovi.es (M.D.)

**Keywords:** birch sap, edible films, antioxidant, photoprotective, active packaging, curcumin

## Abstract

Birch sap consists of a natural water-based solution with valuable compounds such as minerals, sugars, organic acids and phenolic compounds that can be used advantageously in the preparation of edible films. In this study, gelatine- and casein-based films were prepared using birch sap as biopolymer solvent and source of bioactive compounds with the aim of developing new bioactive materials for food packaging. The physical, mechanical, barrier, antioxidant and iron-chelating properties of the obtained films were investigated. Birch sap enhanced the mechanical properties of the films by increasing puncture strength and flexibility, as well as their ultraviolet–visible light barrier properties. In addition, the presence of bioactive compounds endowed the birch sap films with an antioxidant capacity of almost 90% and an iron-chelating capacity of 40–50% with respect to the control films. Finally, to test these films as food packaging material, a photosensitive curcumin solution was packed and exposed to ultraviolet light. Tested films were able to protect curcumin against photodegradation, and the presence of bioactive compounds inside the birch-sap-enriched materials offered an additional 10% photoprotective effect compared to control films. Results showed the potential of birch sap as an environmentally friendly biopolymer solvent and plasticizer that can improve the mechanical and photoprotective properties of the prepared materials.

## 1. Introduction

Since the 1950s the commercial production and consumption of plastics has grown extraordinarily, reaching an annual production of 330 million metric tonnes for 2016, which is estimated to double in the next 20 years [1,2]. The main market for plastic is packaging, a short-term application in which most packaging containers are made up of fossil and non-biodegradable polymers [3]. The spread of petroleum-based plastics in this area and many others is due their numerous advantages, including good mechanical and barrier characteristics, relative low production cost and lightness [3]. Nevertheless, these materials have several disadvantages; since they are synthesized from non-renewable resources and they are non-biodegradable, their disposal makes a serious contribution to environmental pollution [4]. For this reason and in line with the current trends of a sustainable economy, many researchers around the world are focussing on the development of new biodegradable polymeric materials from natural resources such as proteins, lipids and polysaccharides [2,5]. Due to their wide range of chemical functionalities, proteins are considered to be excellent polymers for the development of edible films and biodegradable packaging to improve food product shelf life and food quality [6].

Two proteins extensively studied and used for the preparation of edible films are gelatine and casein [7]. Both caseins and gelatines are desirable hydrocolloids for the development of films, due to their nutritional profile, water solubility, water-binding ability, emulsifying capacity and their good barrier properties to oxygen and other nonpolar molecules, making them promising candidates for food packaging [2,8]. However, the utilization of these types of biopolymers in food packaging has certain limits, as they have poor thermal and mechanical properties compared to petroleum-based materials [2,8]. In order to solve these problems and enhance the workability, elasticity and flexibility of films, plasticizers are usually added to film-forming solutions [9,10].

Plasticizers are low molecular weight, non-volatile molecules that are incorporated into biopolymers to allow the modification of their functional activities by the interruption of hydrogen bonding along the protein chains in the high molecular-mobility amorphous region [8,9]. Among plasticizers, petroleum phthalate derivates are the ones most in demand and with the highest international production; nevertheless, their distribution and use are being restricted because of associated hazards to human health and the environment, due to their high migration tendency. In this context, the development of non-toxic and biodegradable natural-based plasticizers is an area which has been attracting the attention of researchers in recent years [11,12]. In the case of gelatine and casein, many researchers have focussed on the use of biobased plasticizers like polyols such as glycerol, sorbitol, mannitol and xylitol, and monosaccharides such as glucose, mannose, fructose and sucrose [13]. Additionally, to enhance the functional properties of these edible films, some authors have employed different plant extracts rich in phenolic compounds such as anthocyanins, epigallocatechin gallate, ginger essential oil and tarragon essential oil; these compounds enhance the mechanical properties of films while at the same time imparting an antioxidant activity, making them a suitable material for the development of active food packaging. In this regard, a novel and interesting additive to consider when developing bioactive materials is birch sap. Although birch sap has been widely used as a nutritional drink and a cosmetic and nutraceutical ingredient, it can also be considered a by-product of the logging and wood pulp industries. The sap consists of a solution of water with mineral ions, proteins, simple sugars, organic acids and phenolic compounds [14,15]. While its characteristics as an aqueous solution make it an interesting option as an alternative biopolymer solvent, the presence of sugars, proteins and phenolic compounds also enables it to act as a polymer plasticizer and antioxidant additive [15].

Therefore, the aim of this research is to study the effect of birch sap as a biopolymer solvent and source of bioactive compounds on the physical and functional properties of the gelatine and casein materials prepared. For this purpose and for the first time, gelatine and casein bioplastics were prepared and characterized using birch sap as solvent. In addition, the prepared active films were tested as food packaging materials using a simulated food liquid enriched in curcumin, a highly photosensitive compound usually employed by the nutraceutical and food industry.

## 2. Materials and Methods

### 2.1. Birch Sap Samples

Birch sap samples (La Savia) were kindly donated by Renastur Celtibérica S.A.T. (Asturias, Spain). Sap samples were harvested in spring at 1600 m altitude from the high mountain forest of Teverga, in the Rural Area of El Páramo in the Natural Park of Las Ubiñas-La Mesa (43°06′59″ N, 5°57′44″ W) (Biosphere Reserve, Asturias, Spain).

### 2.2. Compositional Analysis of Birch Sap

Although birch sap composition varies with season, latitude and birch strains, it is known that it contains different type of sugars, salts and bioactive molecules, including organic acids, vitamins and phenols such as tannins [15]. To perform the compositional analysis of samples, fresh microfiltered birch sap, without any thermal treatments, was used. After harvesting, each birch sap sample was filtered to eliminate impurities and stored in glass bottles at 4 °C.

#### 2.2.1. Carbohydrate Content

Total carbohydrate content of birch sap samples was measured by the phenol–sulphuric acid method, using glucose as a standard [16]. Briefly, 1 mL of sap was mixed with 0.5 mL of 5% phenol solution and, immediately afterwards, with 2.5 mL of 96% sulphuric acid. After shaking, the mixture was left to stand for 30 min until the absorbance was measured at 492 nm.

#### 2.2.2. Reducing-Sugar Content

Reducing-sugar content was determined by the 3,5-dinitrosalicylic acid (DNS) method, using glucose as the standard [17]. Briefly, 0.5 mL of birch sap was mixed with 0.5 mL of DNS reagent and heated in a boiling water bath for 5 min. Then, samples were cooled with ice for 5 min and 5 mL of distilled water was added. Finally, absorbance was measured at 540 nm, and the total reducing-sugar content was calculated using a calibration curve prepared with serial dilutions of glucose.

The DNS reagent was prepared by dissolving 0.8 g of NaOH, 15 g of sodium potassium tartrate and 0.5 g of DNS in distilled water, up to 50 mL of solution.

#### 2.2.3. Protein Content

Total protein content was determined by a modification of the Lowry method [18]. Briefly, 1 mL of sap sample was mixed with 5 mL of reagent C1 and incubated for 15 min in the dark. Then, 0.3 mL of Folin–Ciocalteu reagent (1:2 in distilled water) was added and left to stand for 30 min in the dark. Finally, absorbance was measured at 580 nm. The calibration curve was prepared with serial dilutions of bovine serum albumin (BSA), and protein content was expressed as mg BSA/g dry extract.

The C1 reagent was prepared by mixing reagents A (2% Na_2_CO_3_ and 0.1 M NaOH), B1 (1% CuSO_4_·H_2_O) and B2 (2% potassium sodium tartrate) in the proportion 50:0.5:0.5 (volume).

#### 2.2.4. Phenolic Compound Content

Total phenolic content was estimated by the Folin–Ciocalteu colorimetric assay described by Martínez-Sanz et al. [19]. Briefly, the Folin–Ciocalteu reagent was diluted 1:10 with distilled water, and 1 mL of the final solution was mixed with 0.2 mL of samples at room temperature. Then, 0.8 mL of sodium carbonate (75 mg/mL) was added, and samples were heated to 50 °C for 30 min. Sample absorbance was read at 750 nm. The calibration curve was prepared using gallic acid as the standard, and the phenolic content was expressed as mg of gallic acid (GA)/g extract.

#### 2.2.5. Salt Composition Analysis

Birch sap salt and metal composition (Ca, Cu, Cr, Se, Fe, Zn, P, K, Mn, Mg) was determined by means of an ICP-MS (Agilent 7700x, Agilent Technologies, Santa Clara, CA, USA), equipped with an integrated I-AS autosampler. The analysis was performed at a nebulizer gas flow of 1.02 L/min and the collision/reaction cell used 4.3 mL/min of He and a kinetic energy discrimination of 3 V to eliminate the interferences. Standards were used for the quantification the metals and salts, which were measured in triplicate and represented as the mean of the replicates ± standard deviation.

#### 2.2.6. Sugars and Organic Acid Analysis by HPLC

Birch sap sugars and organic acids were measured by HPLC (Agilent Technologies 1200 Series), employing an ICSep ICE-ION-300 column (Teknokroma, Sant Cugat del Vallès, Spain) as the stationary phase and H2SO4 (0.45 mM) as the mobile phase (isocratic gradient), at a flow rate of 0.3 mL/min. Mobile phase pH was adjusted to 3.2–3.3 before running the HPLC. A refractive index detector was used to determine the presence of sugars and organic acids. Standard curves of oxalic, maleic, pyruvic, lactobionic, malic, lactic, formic, acetic, propionic, citric, succinic and phosphoric acids were used for the proper identification and quantification of these acids, and standard curves of glucose, fructose and saccharose were used for the proper identification and quantification of sugars.

### 2.3. Concentrated Birch Sap Solution

In order to achieve a three times more concentrated birch sap solution, 750 mL of birch sap was previously cooled to −80 °C for 24 h and then freeze-dried over 48 h to remove water and concentrate the bioactive molecules present in the product. Once lyophilized, the remaining solids were resuspended in 300 mL of fresh birch sap. The use of more highly concentrated birch sap solution resulted in a worsening of the mechanical properties of the obtained films.

### 2.4. Preparation of Gelatine and Casein Films with Concentrated Birch Sap

The film-forming solutions were prepared by dissolving gelatine (gelatine from porcine skin, G1890, Sigma-Aldrich, Darmstadt, Germany) or casein (casein from bovine milk, C-7078, Sigma-Aldrich, Darmstadt, Germany) directly in the birch sap solution with 40% (*w*/*w* of protein) of glycerol. The final concentration of these proteins in the film-forming solution was 5% (*w*/*v*), dissolved by continuous magnetic stirring at 40 °C and 65 °C for gelatine (G) and casein (C), respectively. Control films were formed by dissolving casein (CC) and gelatine (CG) in distilled water instead of sap. Finally, 15 mL of every film-forming solution was poured into a Petri dish of 8.9 cm diameter, in such a way that 0.24 mL of solution was cast per cm^2^ of Petri dish surface.

Birch sap (C, G) and control (CC, CG) films, still in their Petri dish moulds, were dried in an oven at 40 °C for 48 h and then completely removed from the dishes and stabilized in a humidity chamber (HCP50, Memmert, Germany) at 25 °C and 50% humidity for 2 days.

### 2.5. Physical Properties of Birch Sap Gelatine and Casein Films

#### 2.5.1. Light Transmission and Transparency

The barrier properties of films against ultraviolet and visible light were assessed according to the methodology described by Weng et al. [20]. Film transparency was calculated according to Equation (1):

Transparency = *A*_600_/*x* (1)

where *x* is the film thickness in mm and *A*_600_ is the absorbance of the film at 600 nm. Film thickness was measured in six different areas with a micrometer (Mitutoyo, Kawasaki-shi, Japan), with a precision of ±1 µm.

#### 2.5.2. Colorimetric Properties

Film colour and colorimetric properties were measured using an LC 100/ SV 100 spectral colorimeter (Lovibond, UK), as described by Carpintero et al. [21].

The total colour difference (∆E*) was calculated using Equation (2), where ∆L*, ∆a* and ∆b* are the difference values of the corresponding colour parameters, i.e., L* value (lightness), a* value (redness/greenness), and b* value (yellowness/blueness), between films with birch sap and the control films [22]. The Whiteness Index (*WI*), the Chroma and the Yellowness Index (*YI*) of each film were calculated with Equations (3)–(5), respectively [22,23,24]. Each measurement was carried out in triplicate.

(2) $$\text{∆}E*=\sqrt{{\left({∆L}^{*}\right)}^{2}+{\left({∆a}^{*}\right)}^{2}+{\left({∆b}^{*}\right)}^{2}}$$

(3) $$WI=100-\sqrt{{\left(100-L\right)}^{2}+a^{2}+b^{2}}$$

(4) $$Chroma=\sqrt{a^{2}+b^{2}}$$

(5) YI=(142.86×b)/L

#### 2.5.3. Mechanical Properties

The mechanical properties of the gelatine and casein films obtained using birch sap as polymer solvent were analysed following the methodology described by Carpintero et al. [21] using a puncture test using a TA.XT.Plus Texture Analyser (Stable Microsystems, Godalming, UK), equipped with a 5 kg load cell and a 5 mm diameter probe (P/5S), in such a way that films were subjected to a diametral extension.

The puncture resistance (*PS*) and puncture deformation (*PD*) values for each film were calculated using Equations (6) and (7), respectively:

(6) $$PS=\frac{Fm}{Th}$$

(7) $$PD=(\sqrt{D^{2}+R^{2}}-R)/R$$

where *Fm* (N/mm^2^) is the maximum force applied before film rupture, *Th* is the thickness of the film, *D* is the distance covered by the probe from the time it contacts the film until it breaks, and *R* is the radius of the hole created by the probe when breaking the film [20].

#### 2.5.4. Water Vapour Permeability and Solubility

The water vapour permeability of films was tested according to the methodology used by Weng et al. [20]. The water vapour transmission rate (*WVTR*) and the water vapour permeability were calculated according to Equations (8) and (9):

(8) WVTR=G/(t×A)

(9) PWVP=(WVTR×Th)/∆P

where *G*/*t* is the change in the cup weight per unit of time (g/h), which is calculated as the slope of the graphical representation of weight loss versus the time, *A* (m^2^) is the area of the cup covered by the film, *Th* (mm) is the film thickness and ∆*P* (kPa) is the difference in partial vapour pressure between the two sides of the film.

The solubility measurement was also performed according to the methodology described by Weng et al. [20] with some modifications. Briefly, to determine the dry weight of films, gelatine and casein films were cut into circles of 2 cm diameter and dried in an oven at 90 °C for 24 h. Other intact film fragments were immersed in 0.1 M Trizma buffer solutions (Sigma-Aldrich, Darmstadt, Germany) at pH 5, 7 and 9 at room temperature for 24 h. Finally, undissolved film pieces were recovered, dried at 90 °C for 24 h and weighed. The solubility of films was calculated with the following equation:

(10) $$S\;\left(\%\right)=\left(m1-m2\right)/m1\times 100$$

where *S* (%) is the percentage of solubilised film, *m*1 is the initial dry weight of the film, and *m*2 is the dry weight of the remaining film pieces after solubilization, in grams.

#### 2.5.5. Scanning Electron Microscopy (SEM)

A JSM-6610LV scanning electron microscope (JEOL, Peabody, MA, USA) was used to study the microstructure of the transverse section of the birch sap gelatine and casein films. Briefly, film samples were cut into square pieces of 1 × 1 cm using a surgical blade and attached to metal bases using a double-sided adhesive carbon strip. The films were then gold-coated under an argon atmosphere. Film cross-sections were observed at magnifications between 400× and 800×, and with the voltage set at 20 kV.

### 2.6. Antioxidant Activity

The free radical scavenging activity of fresh birch sap samples and birch sap gelatine and casein films was analysed using the DPPH free radical method, following the methodology described by Carpintero et al. [21] with some minor modifications. Briefly, a stock solution of DPPH in ethanol was prepared by mixing 16 mg of DPPH (1805, Cayman Chemical Company, Ann Arbor, MI, USA) with 40 mL of the solvent. Then, 0.6 mL of birch sap was mixed with 0.6 mL of DPPH solution and 4 mL of ethanol; the control was prepared using distilled water instead of sap. To determine the antioxidant capacity of birch sap films, they were cut into 1 × 2 cm strips and placed in glass tubes, to which 15 mL of ethanol and 1 mL of the prepared DPPH stock solution were added. Bottles were stored in darkness, and the liquid absorbance at 517 nm was measured at 15 min for fresh birch sap and at 0.5, 4, 6, 8 and 24 h for casein and gelatine films. After each measurement, the liquid taken to perform the absorbance measurement was returned to the tubes in order to keep the initial volume constant. The antioxidant activity of films was calculated according to Equation (11):

(11) $$Antioxidantcapacity\left(\%\right)=\left({Abs}_{c}-{Abs}_{s}\right)/{Abs}_{c}\times 100$$

where *Abs_c_* is the absorbance at 517 nm of the control, distilled water or casein and gelatine films; and *Abs_s_* is the absorbance at 517 nm of the sample, fresh birch sap and sap casein and gelatine films [23].

### Release Kinetics of Antioxidants

To evaluate the release mechanism for antioxidants from the birch sap films, four different kinetic models were considered to fit the experimental data [25,26]. Model 1 is given by the zero-order equation:

(12) $$\frac{M_{t}}{M_{inf}}=k_{0}t$$

where $M_{t}$ and $M_{inf}$ are the cumulative antioxidants released at time *t* and infinite time, respectively; $k_{0}$ is the zero-order release constant; and t is the release time.

Model 2 is the first-order kinetic model:

(13) $$\frac{M_{t}}{M_{inf}}=1-e^{-k_{1}t}$$

where $k_{1}$ is the first-order release constant.

Model 3 is the Higuchi model, which is probably the most famous and often used mathematical equation to describe the release mechanism of an active compound from matrix systems, frequently thin-film hydrogels:

(14) $$\frac{M_{t}}{M_{inf}}=k_{H}t^{0.5}$$

where $k_{H}$ is the Higuchi release constant.

Finally, Model 4 is given by the Ritger–Peppas equation, which is expressed by the following equation:

(15) $$\frac{M_{t}}{M_{inf}}=kt^{n}$$

where k is the release rate constant, which depends on the structural and geometrical characteristics of the release system, and n is the diffusional exponent, which indicates the drug-release mechanism.

### 2.7. Ferrous Ion Chelating Capacity

The ferrous ion chelating ability of fresh birch sap and birch sap films was assessed according to the method of Decker et al. [27]. Briefly, 5 mL of the sample solutions and 0.3 g of the birch sap gelatine and casein films were mixed with 0.1 mL of 2 mM FeCl_2_ and 0.2 mL of 5 mM ferrozine solution. After incubation at room temperature for 10 min, the absorbance was measured at 562 nm. The Fe^2+^/ferrozine complex has a high absorbance at this wavelength, so high chelating ability is shown as a low absorbance. The chelating ability as a percentage was calculated as follows:

(16) $$Ferrous\;chelating\;ability\left(\%\right)=(A_{blank}-A_{t}/A_{blank})\times 100$$

where $A_{t}$ is the absorbance of the test sample, fresh birch sap and sap gelatine and casein films, and $A_{blank}$ is the absorbance of the control, water or control gelatine and casein films.

### 2.8. Film Application as Active Packaging

#### Film Application as Active Pouches to Pack Curcumin Solution

In order to test the capacity of the birch-sap-enriched films to protect photosensitive food liquids, rectangular pouches were made from gelatine and casein films with a conventional plastic sealer. In this case, a curcumin solution was selected to test the photoprotective properties of films, since curcumin is a food-grade bioactive compound easily oxidized by UV and visible light. For that purpose, a 50 µM curcumin stock solution was prepared by dissolving curcumin in absolute ethanol; the mixture was left in agitation for 1 h, completely protected from sunlight. Pouches loaded with 8 mL of the curcumin solution were exposed to UV light at 313 nm for 4 h to produce the oxidation of the curcumin and thus evaluate the protective effect of the pouches. To study the degradation of curcumin, samples were taken after 15, 30, 60, 120, 180 and 240 minutes, and the UV-vis absorption spectra of the curcumin solution in the 250–600 nm range was recorded with a UV-Visible Genesys 150 spectrophotometer (Thermo Scientific, Waltham, MA, USA), using quartz cells with an optical path of 1 cm. As positive control, 15 mL of the curcumin solution in an open petri dish was subjected to the same oxidative conditions.

### 2.9. Statistical Analysis

Each different experiment was performed in duplicate, and the average and the corresponding standard deviation of the results obtained are represented in each case. In order to determine the significant differences between the film samples, a simple analysis of variance (ANOVA) with a confidence level of 95% was performed. These analyses were performed using the statistical software Statgraphics Centurion XVI (version 16.1.11).

## 3. Results and Discussion

### 3.1. Birch Sap Composition

The major components of fresh *Betula pendula* sap are presented in Table 1. According to the literature, the protein concentration of birch sap is commonly between 0.003 and 0.06 g/L [28], although higher concentrations of between 0.12 and 0.27 g/L have also been reported [14,29]. However, as can be observed, the concentration of protein in the fresh sap samples was higher than those reported to date, which can be explained by the fact that the birch species usually studied are *Betula platyphylla* or *Betula verrucosa*, while in this study, samples were from *Betula pendula*. In addition, the time of the year when samples are collected has a major influence on the chemical composition of the birch sap, and in this study, the sap was collected at the end of spring, when the protein concentration is particularly high due to the presence of a higher nitrogen concentration in the xylem [28]. With regard to the concentration of carbohydrates and simple sugars, the greater part of total carbohydrates were simple sugars, glucose and fructose, while complex sugars constituted only 11.1% of the total. The amount of simple sugars, 0.54 g/L glucose and 3.50 g/L fructose, was lower than those reported in other studies to date, i.e., 5.39 g/L fructose and 4.46 g/L glucose, which may be due to the stage of exudation period when sap was collected [29]. Although lower than in other studies, this relatively high concentration of simple sugars suggests that the fresh birch sap could be used as a plasticizer for common biopolymers in the food packaging industry, since glucose and fructose have already been successfully tested as edible film plasticizers by other authors [30,31].

In addition, the concentration of phenolic compounds detected in the birch sap was slightly higher than the range of 0.035 to 0.055 g/L reported by Grabek-Lejko et al. [14]. It should be underlined that the phenolic compounds are closely related to the antioxidant and chelating activity shown by the fresh sap, and they also affect the physical properties of polymers, especially polysaccharides, by favouring their aggregation [15,32]. Additionally, also detected in the analysed birch sap samples were acids, such as oxalic acid (12.3 min), succinic acid (27.97 min), formic acid (28.8 min), acetic acid (33.2 min) and propionic acid (Table 1), whose concentrations are similar to those reported by other authors in other saps, such as palm sap [33]. The presence of these molecules is interesting because all of them have been reported as antibacterial, anti-inflammatory and antioxidant compounds [34,35].

Furthermore, the micronutrient profile, especially minerals, of the fresh birch sap was analysed (Table 1). It can be observed that birch sap was rich in microelements like calcium (Ca), magnesium (Mg), manganese (Mn), potassium (K), phosphorus (P) and zinc (Zn), with small amounts of iron (Fe), copper (Cu), chromium (Cr) and selenium (Se). Keita et al. [15] demonstrated that these minerals present in the birch sap, together with phenolic compounds, caused an increase in the flow consistency of the gels in which they were included. This phenomenon is produced due to all these compounds, which favour the intermolecular interactions between chains of biopolymers by facilitating electrostatic interactions or by decreasing electrostatic repulsions. In this regard, birch sap could be a valuable ingredient in the preparation of edible films, as it could increase the strength and homogeneity of the materials produced.

According to the results obtained from the compositional analysis of the birch sap collected, this product could be an interesting aqueous-based solvent for hydrophilic polymers. In this case, gelatine and casein films were prepared by dissolving these proteins directly in the birch sap and their physical and mechanical properties were assessed.

### 3.2. Physical Properties of Birch Sap Gelatine and Casein Films

#### 3.2.1. Visual Aspect, Light Transmission and Transparency

**Figure 1:**
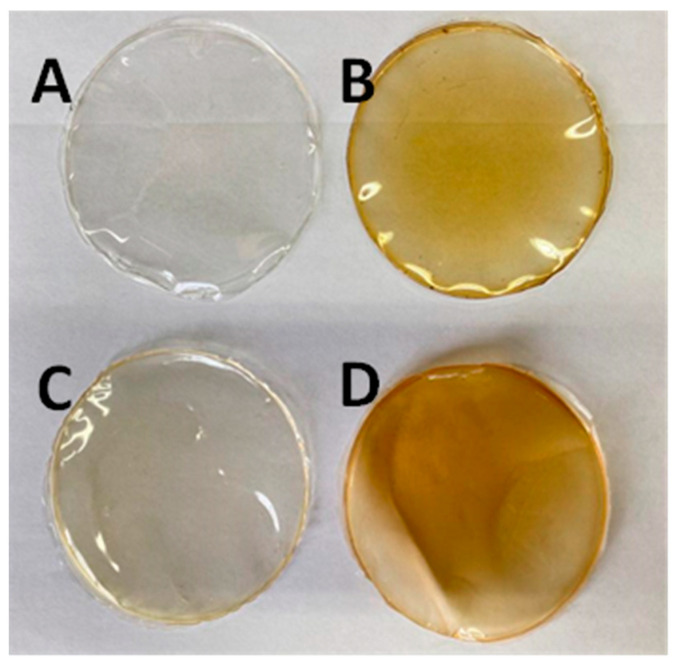
Visual aspect of control gelatine film (**A**), birch sap gelatine film (**B**), control casein film (**C**) and birch sap casein film (**D**).

Both the control films prepared by dissolving gelatine or casein in distilled water and films prepared using fresh birch sap as solvent were easily removed from the Petri dishes. Under visual inspection, films with birch sap were as homogeneous as the control ones, significantly more opaque and with a slight orange-brown coloration, and those prepared with casein were less transparent than the gelatine ones (Figure 1).

Light transmission properties of films were evaluated at wavelengths from 200 to 800 nm, and the transmittance values are shown in Table 2. As sunlight is a strong oxidizing agent for lipids and other photosensitive molecules present in food, cosmetics and other products, low transmittance is a crucial property for barrier materials to prevent the degradation of these compounds [21]. Both casein and gelatine control films showed significant UV/Vis light barrier properties, with those of casein films being higher than those of gelatine, which may be because of the lack of tryptophan in gelatine and its low content of phenylalanine and tyrosine [36,37]. The use of birch sap as solvent for these biopolymers resulted in films with better light-barrier properties than casein and gelatine films prepared using water as the solvent, which suggests that the biomolecules present in the sap endow gelatine films with the ability to act as a barrier against ultraviolet light (200–400 nm). This decrease in UV/Vis light transmittance values for the casein-based films prepared with birch sap could be explained by the fact that during the drying of the film-forming solution, the compounds present in the birch sap produced conformation changes in the native casein structure different from those produced in the presence of water. In this regard, the phenol groups and the aromatic chromophores present in proteins, such as those found in the amino acid residues of phenylalanine, tyrosine, and tryptophan, may interact with the simple sugars in birch sap producing protein conformational changes, increasing the exposure of these amino acids from the inside of the protein structure to the exterior environment, increasing the absorbance of the packing materials in the UV/Vis region [38,39]. As with transmittance, the use of birch sap as a solvent affected the transparency of casein films, which decreased (Table 2), augmenting their barrier properties as predicted above.

#### 3.2.2. Colorimetric Properties

The colour attributes of the gelatine and casein films prepared using birch sap as the solvent are shown in Table 2. The incorporation of birch sap molecules conferred an orangish colour to the casein films, which corresponded to a decrease in the L* and an increase in the *YI* and b* values. In the case of gelatine films, the presence of birch sap did not have any noticeable effect on the L* or a* of the films, but it did produce a significant decrease in the values of the b* and *YI* parameters, which means an increase in the blue colouration of films. These differences could be a result of the colour contribution of polymerized quinone, the product of phenolic–protein interaction, and this interaction depends on the types of proteins and phenolic compounds present in the medium, which are different for gelatine and casein [40].

In the case of the total colour difference (∆E*) between the films with birch sap (G and C) and the control gelatine and casein films (CG and CC), it can be observed that the values for this parameter were higher than 3.0, which indicates that the colour changes produced in the material due to the addition of birch sap would be appreciated by the human eye. These ∆E* results agree with chroma values, i.e., the total amount of colour, which increase with the use of birch sap as solvent.

#### 3.2.3. Mechanical Properties

The mechanical properties of gelatine and casein films obtained using birch sap as the biopolymer solvent are shown in Table 3. In terms of film thickness, a tendency to decrease was observed when using birch sap as the solvent instead of distilled water, an effect that was more noticeable in the case of casein films than in the gelatine ones. These results may be due to the way birch sap compounds interact with the aromatic groups of gelatine and casein proteins, modifying their conformational structure and, therefore, the microstructure of the film matrix [38,39].

Regarding the mechanical properties, the use of birch sap as the solvent had a noticeable effect on the films’ puncture deformation (PD) and puncture strength (PS) (Table 3). PD values showed an important increase in both types of films, casein and gelatine, when birch sap was used as the solvent. Therefore, the birch sap molecules in the edible films acted as a plasticizer, making the films more elastic. Overall, the results agree with those obtained by Scartazzini et al. [41] and Tongnuanchan et al. [42], who observed that adding small amounts of oils, such as palm oil and essential mint oil, resulted in an increase in the elasticity of gelatine films; lipids present in these oils act by dispersing uniformly within the matrix of the films, decreasing protein–protein interactions. In the case of birch sap, the plasticizing properties may be due to the high content of simple sugars like mannose, glucose, fructose, sorbitol, xylitol and mannitol, which reduce the cohesive forces between starch granules and improve the extensibility and flexibility of the obtained films [30,31].

On the other hand, an increase in PS values was also observed, which means that the use of sap as solvent also favoured an increase in the films’ mechanical resistance. Such a change is not typically observed when using other types of plasticizers, which cause an improvement in the elastic properties of the materials by decreasing the number of intermolecular forces along polymer chains and, therefore, their mechanical resistance. In the case of plasticizers like vegetable oils, a decrease in tensile or puncture strength can be observed [41,42], an effect that was also observed by other authors when using glycerol and low molecular sugars as plasticizers, which reduce the crystallinity index and inhibit the formation of intercalated structures [31]. Regarding the chemical composition of birch sap, the increase it caused in the films’ puncture strength may be due to the high content of phenolic compounds, which can be converted to quinone, a protein cross-linker, increasing the number of protein–phenolic cross-linking interactions via hydrogen bonding between hydroxyl groups on the phenolic compounds and the -NH3+ groups of protein amino acids [15,43]. In summary, the results reveal that birch sap as a polymer solvent had a good plasticization effect, improving the flexibility and rigidity of the films.

#### 3.2.4. Water Vapour Permeability (WVP) and Solubility

Among the most interesting and important characteristics of the materials used for food packaging are their barrier and permeability properties with respect to the transfer of water vapour, gases and aroma molecules [44]. The moisture barrier property is an important parameter for food packaging applicability, which is closely related to the type and amount of plasticizers and additives added to the film-forming solution [20,45].

The WVP data for gelatine and casein films obtained using birch sap as the polymer solvent is displayed in Table 3. From these results, it can be observed that in both cases, gelatine and casein, films with birch sap had a higher WVP than the controls, something that may be due to the hydrophilic nature of simple sugars present in sap. These results are in concordance with those of Gao et al. [31], who observed that when 10 or 20% of simple sugars—sucrose, fructose and glucose—were added to starch films, their WVP increased from 4.37 × 10^−10^ to 4.78 × 10^−10^ g/m·s·Pa. Similarly, when Pruneda et al. [46] added oregano extracts to soy protein films, the presence of polar oregano compounds, like phenolic acids and flavonoids, increased the hydrophilic properties of the films. In contrast, Friesen et al. [47] reported that the addition of phenolic compounds to soy protein films decreased vapour permeability due to the increase in matrix compactness as a result of cross-linking reactions. Here, it can be seen that the hydrophilic nature of the simple sugars and phenolic compounds has a higher effect on film barrier properties than the matrix compactness.

Solubility of edible films in water is one of the most important parameters to take into account when selecting their final application in industry. While some applications require high solubility for the film to be easily dissolved before consumption, others need low water-soluble properties to guarantee the film’s material resistance [48]. The solubility values at different water pH values of the films are shown in Table 3. It can be observed that casein is totally soluble at all pH values, while gelatine solubility increases with increasing pH—more alkaline solvents. In the case of gelatine films, solubility values also increase in films formed using birch sap as the solvent for the biopolymer, which may be due to the high concentration of simple sugars, compounds with a high solubility in water which give the polymer molecules a higher affinity to attract water. These results are in concordance with those obtained by Gheribi et al. [49], who observed that by adding low molecular weight plasticizers—glycerol or other polyol plasticizers—to edible films, their water solubility was significantly increased.

#### 3.2.5. Microstructure

**Figure 2:**
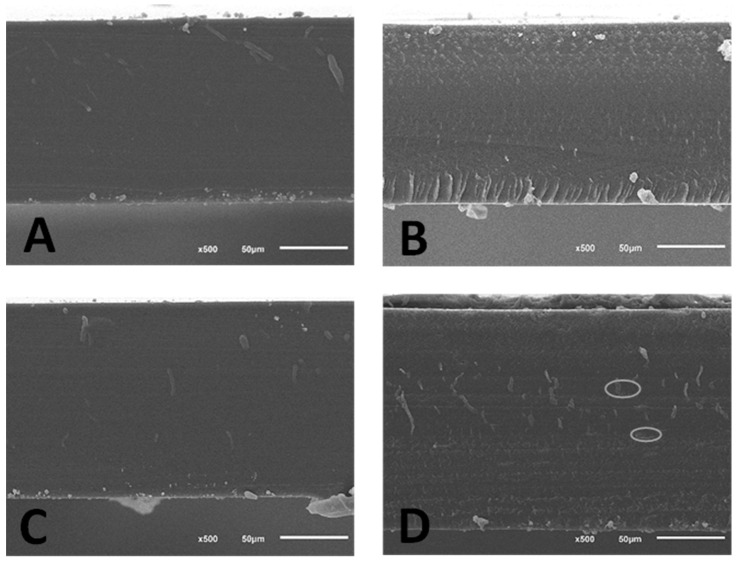
Micrographs of gelatine and casein films using birch sap as the solvent for the biopolymer. (**A**): Gelatine film without birch sap. (**B**): Casein film without birch sap. (**C**): Gelatine film with birch sap as solvent. (**D**): Casein film with birch sap as solvent. In micrograph (**D**) the ovals indicate the presence of micropores in the matrix.

SEM micrographs representing the morphology of the cross-sections of gelatine and casein films obtained using birch sap as biopolymers solvent are depicted in Figure 2. Micrographs reveal that all films showed a highly smooth, compact and continuous film matrix, independently of the solvent used, distilled water or birch sap. However, some slight differences can be seen between control and sap films. In the case of gelatine, a layer arrangement appeared when using birch sap as the solvent; this type of phenomenon is observed when using plasticizers like glycerol and sorbitol and is a possible explanation for the increase in the films’ WVP and solubility. On the other hand, casein films with birch sap are less homogeneous than those made with distilled water; holes in the matrix can be seen, which may be due to poorer incorporation of plasticizers than in the gelatine matrix, where there are no pores due to the total compatibility of the plasticizer with the biopolymer [7,49].

#### 3.2.6. Antioxidant Activity and Chelating Capacity

**Figure 3:**
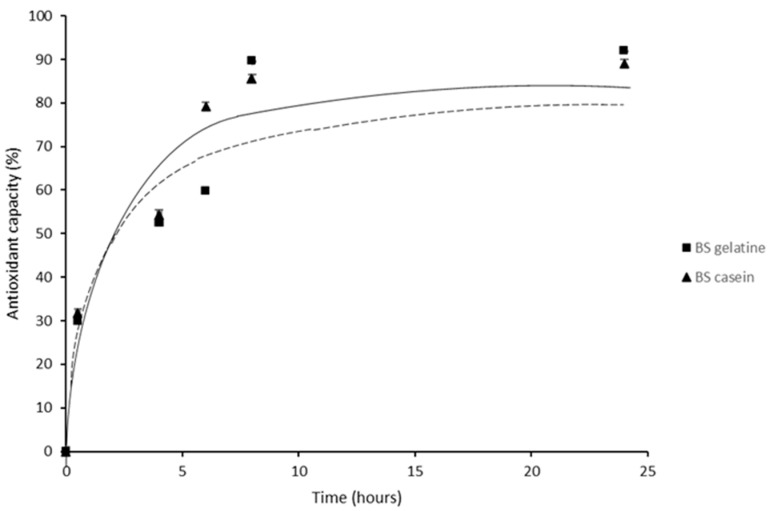
DPPH radical neutralizing effect of birch sap gelatine (solid line) and casein (dashed line) films during the first 24 h of study.

Figure 3 shows the antioxidant capacity of the birch sap gelatine and casein films. As can be seen from the graph, in both types of films, gelatine and casein, the use of birch sap as a biopolymer solvent gives them an important antioxidant capacity as compared to those made with distilled water. Moreover, the antioxidant activity increases steadily until 8 h, when it reaches the maximum, almost 90% of antioxidant capacity in both cases, which suggests that at that time most of the antioxidant compounds retained in the gelatine and casein matrix had been released. This antioxidant capacity may be due to the presence of different types of phenolic compounds and organic acids contained in the birch sap; phenolic acids are molecules with greater antioxidant capacity due to their radical scavenging activity via hydrogen atom donation, electron donation or singlet oxygen quenching [50].

Regarding the chelating capacity, the use of birch sap as biopolymer solvent made it possible to obtain films with an important capacity to chelate iron (II), namely 41.29 ± 0.01% for gelatine films and 59.60 ± 0.00% for casein films. This high chelating capacity is likely due to the presence of organic acids and proteins or amino acids in birch sap films [51,52]. According to these results, the addition of birch sap to the casein and gelatine films results in bioplastics with antioxidant and chelating properties without the presence of synthetic additives.

#### 3.2.7. Antioxidant Release Kinetics

The mathematical modelling of bioactive compound release is important in order to analyse the release behaviour and evaluate the performance of the active packaging, as it allows the diffusion coefficients of the bioactive compounds to be known. The release kinetics of birch sap antioxidants from casein and gelatine films were evaluated by fitting the release data shown in Figure 3 to four mathematical models (zero-order, first-order, Higuchi and Ritger–Peppas model). The kinetic parameters and the correlation coefficients (R^2^) with respect to time are presented in Table 4.

The release of antioxidants from films fits well to all the used models, with the best statistical fit being the Higuchi model for both types of films, casein and gelatine. This best fit to the Higuchi model indicates that the release model is diffusional, which was verified when the experimental data were fitted to the Peppas–Sahlin model. In this sense, Peppas–Sahlin fitting reveals that antioxidant release is case II (*n* ≥ 0.5), which can be described as a combination of Fickian diffusion and polymer chain dissolution and relaxation [53]. In case II relaxation, the release rate of antioxidants corresponds to zero-order release kinetics, release is only dependent on time, and the liberation rate is constant, in agreement with the reasonable fitting to the zero-order model (R^2^ = 0.95) [54]. The fitting to the first-order kinetic model suggests that the release rate of the antioxidants from the casein films is partially concentration-dependent; however, for gelatine films, the low first-order fitting value obtained suggests that the concentration of antioxidants is not a controlling variable. Furthermore, for all models tested, except for the Ritger–Peppas model, *k* values were slightly higher for casein than for gelatine, showing that the release of active compounds is faster in the casein films than in the gelatine ones.

According to the results of the kinetic analysis, the mechanism of antioxidant release from gelatine and casein films could be divided into two stages: the first stage is a passive diffusion of antioxidants in contact with the solvent or near the surface of films; and the second part involves the relaxing stage in which films absorb solvent and swell, leading to the amplification of diffusion spaces [55].

#### 3.2.8. Film Application as Active Packaging: Packing Curcumin Solutions

According to the results obtained, the relatively high antioxidant and barrier properties of the films suggest that they could be of interest for the preparation of active packaging materials for photosensitive food-related bioactive compounds. In order to study the protective capacity of these films, the photodegradation of curcumin when UV light (314 nm) was incident on gelatine and casein bags loaded with an ethanol solution containing curcumin was investigated. Curcumin, the main component of *Curcuma longa*, is a lipophilic polyphenolic antioxidant compound whose importance in food science has grown significantly in recent years due to its potential as a natural antioxidant in beverages and functional foods [56]. However, its chemical instability and photodegradation limit its use in the industry [56].

**Figure 4:**
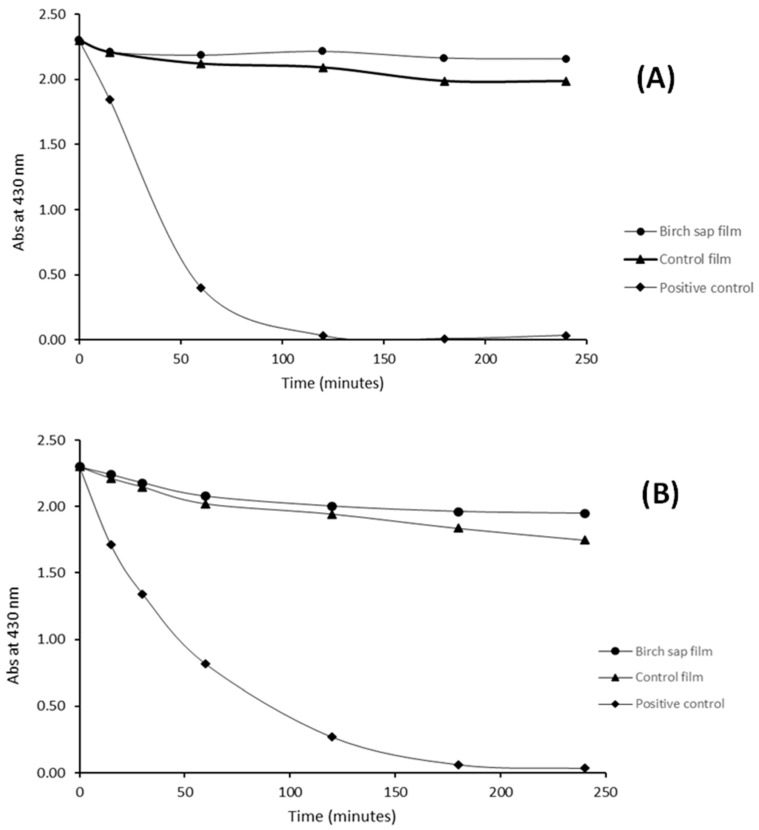
Kinetic degradation of curcumin after exposure to UV source lamp at 313 nm during 4 h. (**A**) Casein films. (**B**) Gelatine films.

Figure 4 shows the change in absorbance at 430 nm of free curcumin solution (positive control) and of the curcumin solution packed in the control gelatine and casein films and in the birch sap gelatine and casein films. It can be seen that curcumin was completely degraded after 3 h of exposure to UV light, while the films offer curcumin an important degree of protection against degradation, the maximum degradation of the component being 17.46% in the case of the control gelatine film. It can also be observed that, although gelatine and casein control films offer curcumin important defense against degradation, the curcumin concentration shows a constant decrease with time, while birch sap films afford sufficient protection for the curcumin concentration to reach a plateau and remain almost constant over time. These phenomena may be due to the release of the antioxidants from the film matrix, offering an extra photoprotective effect of approximately 10%, by favouring their own photooxidation instead of that of curcumin. These results are in concordance with those observed by Castillo et al. [57] when encapsulating curcumin in γ-cyclodextrin. It was found that the complexation of curcumin to γ-cyclodextrin prevented the demethoxylation and photochemical degradation undergone by free curcumin.

## 4. Conclusions

For the first time, birch sap was shown to be an efficient solvent and plasticizer for biopolymers, yielding casein and gelatine films with improved physical and bioactive properties. The presence of simple sugars, proteins, ions and phenolic compounds could have interesting nutritional and technological benefits for the preparation of edibles films; particularly, simple sugars caused it to behave like a common plasticizer by improving the elasticity and solubility of the films. In addition, the high number of phenolic compounds in birch sap produced an increase in film compactness and, therefore, mechanical resistance. The presence of these phenolic compounds and other bioactive molecules, such as organic acids, provided the films with important antioxidant, chelating and light-barrier properties, which were tested using a photosensitive compound, curcumin. Results proved that casein and gelatine films obtained using birch sap as the polymer solvent give a protective effect against photochemical degradation of this sensitive compound. Consequently, these birch sap gelatine and casein films could potentially be used as an alternative for packaging food or easily photo-oxidizable compounds.

## Figures and Tables

**Table 1:** Birch sap chemical and mineral composition and antioxidant and chelating capacity.

**Proteins (g/L)**	**Total Carbohydrates (g/L)**		**Simple Sugars (g/L)**		**Phenolic Compounds (g/L)**		**Antioxidant Capacity (%)**	**Iron Chelating Capacity (%)**	
0.32 ± 0.01	7.03 ± 0.32		6.25 ± 0.27		0.09 ± 0.01		28.47 ± 1.40	30.27 ± 0.05	
**Mg (ppb)**	**P (ppb)**	**K (ppb)**	**Ca (ppb)**	**Cr (ppb)**	**Mn (ppb)**	**Fe (ppb)**	**Cu (ppb)**	**Zn (ppb)**	**Se (ppb)**
1.1 × 10^4^ ± 0.5	9.2 × 10^3^ ± 2.1	7.4 × 10^4^ ± 0.2	4.8 × 10^4^ ± 0.6	2.4 × 10^−1^ ± 1.3	5.2 × 10^3^ ± 0.3	9.7 × 10^1^ ± 1.3	3.5 × 10^1^ ± 1.1	1.5 × 10^3^ ± 0.5	<0.000
**Oxalic acid (mg/L)**	**Formic acid (mg/L)**		**Succinic acid (mg/L)**	**Acetic acid (mg/L)**	**Propionic acid (mg/L)**		**Glucose (mg/L)**	**Fructose (mg/L)**	
41.55 ± 5.82	67.27 ± 6.85		584.00 ± 13.47	145.27 ± 23.37	565.50 ± 29.09		542.30 ± 13.34	3495.00 ± 182.86	

**Table 2:** Transmittance, transparency and colour attributes of gelatine and casein films: control gelatine film (CG), birch sap gelatine films (G), control casein films (CC) and birch sap casein films (C).

**Films**	**Transmittance (%)**							**Transparency**
	**200 nm**	**280 nm**	**350 nm**	**400 nm**	**500 nm**	**600 nm**	**700 nm**	
**CG**	0.02 ± 0.00	12.35 ± 1.20	77.24 ± 1.89	86.95 ± 1.48	91.80 ± 1.84	93.50 ± 0.71	93.10 ± 1.70	0.247 ± 0.032 ^a^
**G**	0.01 ± 0.00	0.42 ± 0.11	24.00 ± 3.82	56.55 ± 2.47	81.85 ± 0.49	90.40 ± 0.57	94.80 ± 2.55	0.268 ± 0.038 ^a^
**CC**	0.01 ± 0.00	0.04 ± 0.00	45.45 ± 2.05	72.20 ± 1.56	86.55 ± 0.49	90.85 ± 1.34	92.10 ± 1.41	0.230 ± 0.008 ^b^
**C**	0.00 ± 0.00	0.00 ± 0.00	0.68 ± 0.14	8.27 ± 1.31	30.40 ± 6.08	48.50 ± 6.89	58.35 ± 4.74	1.664 ± 0.215 ^a^
**Film**	***L*** *	***a*** *	***b*** *	***b*** *	∆E*	***WI***	***Chroma***	***YI***
**CG**	91.30 ± 0.14 ^a^	1.70 ± 0.00 ^a^	0.40 ± 0.14 ^a^	0.40 ± 0.14 ^a^	-	91.12 ± 0.13 ^a^	1.75 ± 0.03 ^b^	0.62 ± 0.22 ^a^
**G**	91.35 ± 1.48 ^a^	2.40 ± 0.85 ^a^	−4.65 ± 0.92 ^b^	−4.65 ± 0.92 ^b^	4.98 ± 1.15	89.02 ± 1.88 ^a^	5.20 ± 1.20 ^a^	−7.36 ± 1.57 ^b^
**CC**	91.40 ± 0.70 ^a^	2.55 ± 0.21 ^a^	−1.60 ± 1.70 ^b^	−1.60 ± 1.70 ^b^	-	91.39 ± 0.93 ^a^	1.95 ± 1.22 ^b^	−0.48 ± 1.96 ^b^
**C**	85.65 ± 0.92 ^b^	3.00 ± 0.14 ^a^	12.10 ± 4.53 ^a^	12.10 ± 4.53 ^a^	17.10 ± 2.40	80.77 ± 2.18 ^b^	13.49 ± 4.42 ^a^	21.14 ± 7.33 ^a^

Different letters in the same column between CG and G, and CC and C indicate significant differences (*p* < 0.05).

**Table 3:** Thickness, puncture strength (PS), puncture deformation (PD) and solubility of gelatine and casein films: control gelatine film (CG), birch sap gelatine films (G), control casein films (CC) and birch sap casein films (C).

Film	Thickness (mm)	PS (N/mm)	PD (%)	WVP (g × mm/m^2^ × h × kPa)	Solubility		
					pH 3	pH 7	pH 9
**CG**	0.130 ± 0.019 ^a^	565.56 ± 30.92 ^b^	52.75 ± 1.58 ^b^	1.14 ± 0.04 ^a^	21.51 ± 5.97	35.67 ± 5.72	40.81 ± 11.15
**G**	0.125 ± 0.007 ^b^	691.00 ± 27.75 ^a^	116.62 ± 10.72 ^a^	1.72 ± 0.89 ^a^	67.90 ± 10.00	79.08 ± 2.56	84.07 ± 3.95
**CC**	0.240 ± 0.070 ^a^	236.19 ± 28.93 ^b^	25.46 ± 2.71 ^b^	5.474 ± 0.49 ^b^	100.00 ± 0	100.00 ± 0	100.00 ± 0
**C**	0.133 ± 0.030 ^b^	425.96 ± 17.81 ^a^	69.13 ± 4.32 ^a^	6.76 ± 0.84 ^a^	100.00 ± 0	100.00 ± 0	100.00 ± 0

Different letters in the same column indicate significant differences (*p* < 0.05).

**Table 4:** Parameters of the kinetic mathematical models obtained after fitting the experimental data to the equations proposed by the different kinetic models chosen.

System	Kinetic Models									
	Zero-Order Model		First-Order Model			Higuchi Model		Ritger–Peppas Model		
	*k*_0_	R^2^	*K*_1_	*M_max_*	R^2^	*k_H_*	R^2^	*K*	*n*	R^2^
**Gelatine**	0.187	0.954	0.0036	92.380	0.798	3.721	0.963	1.500	0.678	0.954
**Casein**	0.200	0.951	0.0038	91.600	0.962	3.959	0.990	1.370	0.701	0.946

## Data Availability

Data are contained within the article.
